# Leptin Resistance and the Neuro-Adipose Connection

**DOI:** 10.3389/fendo.2017.00045

**Published:** 2017-03-06

**Authors:** Andreia Barateiro, Ines Mahú, Ana I. Domingos

**Affiliations:** ^1^Obesity Laboratory, Instituto Gulbenkian de Ciência, Oeiras, Portugal; ^2^Faculty of Pharmacy, Research Institute for Medicines (iMed.ULisboa), Universidade de Lisboa, Lisbon, Portugal

**Keywords:** leptin resistance, metabolic syndrome, MONA LISA hypothesis, obesity, sympathetic neurons

Obesity is a public health concern affecting both genders at all ages around the world (1). The worldwide prevalence of obesity is rapidly increasing and has nearly doubled between 1980 and 2016 (2). Consequently, it places a large financial burden on the economy due to the increased morbidity and mortality, as well as the reduced quality of life and development of chronic diseases (3). Obesity is typically characterized by excessive amounts of the hormone leptin, a cytokine-like molecule produced in white adipose tissue (WAT) that is secreted into the systemic circulation (4). The circulating levels of leptin are proportional to the amount of fat and function as the afferent signal in a negative feedback loop that seeks to maintain body fat in a very narrow range of variation (5). Leptin has a central role in body weight homeostasis due to its inhibition of food intake inhibition and stimulation of energy expenditure. The effect of leptin on body weight is attributed to its action in a specific brain region, the hypothalamus. Hence, leptin is released by adipocytes in proportion to the size of fat depots, enters the circulation, and reaches the central nervous system by crossing the blood-brain barrier (BBB) through receptor-mediated endocytosis (6) in which it acts mainly through the arcuate nucleus of the hypothalamus to mediate most of its actions (7). Specifically, leptin modulates the activity of two types of neurons to inhibit appetite, through production of anorexigenic peptides by the pro-opiomelanocortin (POMC) neurons (8) and suppression of the orexigenic agouti-related protein (AgRP) neurons (9). Besides acting on the hypothalamus to suppress appetite, leptin also induces lipolysis in WAT and thermogenesis in brown adipose tissue (BAT) and browning of WAT, *via* the activation of the sympathetic nervous system (SNS) (10). However, in most obese subjects, despite its high serum levels, leptin fails to perform its physiological functions and consequently fails to reduce weight (11, 12). This effect has been coined as leptin resistance.

The concept of leptin resistance is used to define states where hyperleptinemia is combined to lack of response to the hormone, with consequent maintenance of body weight excess and increased food consumption (13). Nevertheless, it has been described that the effect of leptin treatment on the control of body weight through the regulation of both food intake and energy expenditure is differently exerted in lean and obese humans, suggesting different sensitivity to the hormone (14). Although the mechanisms behind the development of leptin resistance are still unclear, several models have been proposed. As the hypothalamus mediates the anti-obesity actions of leptin, one of the models proposed a decrease in leptin transport across the BBB (15). In addition, a more recent study has shown that BBB impairment can also be attributed to higher plasmatic levels of cytokines and fatty acids in obese subjects (16). Another proposed mechanism is the disruption of leptin signal transduction as several proteins are able to inhibit the signaling from cytokine receptors (17, 18). In this context, suppressor of cytokine signaling 3 (SOCS3) seems to be a key protein in central leptin resistance because its loss of function in the hypothalamus confers protection to high-fat diet (HFD)-induced obesity (19). Other studies have also demonstrated that HFD induces SOCS3 expression by leptin in POMC and AgRP neurons (20, 21). More recently, increased endoplasmic reticulum stress was also suggested as a mediator of the obesity-associated central leptin resistance (22). Interestingly, hypothalamic inflammation is also emerging as a key mechanism for leptin resistance development as it can be responsible for structural changes leading to inefficient circuits in food intake control (22–24). As an early response to HFD, reactive glial cells are able to proliferate and acquire a proinflammatory state even within few days before the detection of significant alterations in body weight (25). This early inflammatory response is neuroprotective. However, the following chronic inflammatory state is responsible for synaptic alterations in the hypothalamus that are connected to the loss of leptin responsiveness (26). Moreover, several studies have demonstrated that inflammatory mediators, such as tumor necrosis factor α (TNF-α), interleukin 1β (IL-1β), and C reactive protein, are elevated in obesity (27–29) and are involved in the increase of circulating leptin concentrations in rodents and humans, suggesting that these factors may be related to hyperleptinemia and leptin resistance onset (30, 31). Interestingly, the ablation of IL-1β receptor and TNF-α receptor 1 in mice protects from obesity induced by HFD (32, 33). On the other hand, other studies have already demonstrated that leptin is able to induce the secretion of proinflammatory cytokines (34).

But, when it comes to defining leptin resistance, it seems that our understanding of the involvement of hyperleptinemia in the development of impaired satiety is far more understood than that which is responsible for the loss of the lipolytic action of leptin. So, besides not so studied, a parallel peripheral leptin resistance can occur (35). In fact, elevation of SNS tone in WAT and BAT is essential for the dissipation of energy *via* activation of beta-adrenergic receptors post-synapticaly in target organs (36). Hence, the induction of lipolysis could be a therapeutic target in the context of obesity management, but for that we need to understand both how leptin controls the SNS output to WAT (37) and BAT (38) and how the decrease in energy expenditure observed upon leptin resistance develops, consequently driving the progression of obesity into metabolic syndrome.

The definition of metabolic syndrome, for which leptin resistance is one of the major risk factors, highlights the connection between elevation of metabolic markers (abdominal obesity, high triglyceride, low high-density lipoprotein cholesterol blood concentrations, and hyperglycemia) and elevation of risk for developing cardiovascular diseases (39). Moreover, obesity has been associated with a chronic increase of sympathetic tone, which can explain the development of obesity-associated hypertension and other cardiovascular morbidities. These associations were based on observation of increased urinary noradrenaline, efferent muscle sympathetic nerve activity (MSNA), and noradrenaline spillover (global and regional) to the plasma in obese subjects (40). In the context of positive energy balance, the increase in SNS activity in obese individuals would serve the purpose of counteracting adiposity, by increasing energy expenditure and preventing weight gain (40). However, such increase of SNS tone seems to be differentially distributed across organs, such as heart, blood vessel muscle, or various fat depots. Indeed, abdominal visceral fat volume positively correlates with MSNA, while subcutaneous adipose mass seems not to be correlated with MSNA (41). The heart, kidney and muscle seem to be the major targets of increased sympathetic tone in obesity, linked to the development of hypertension, while limited lipolytic responsiveness to SNS-mediated stimuli in WAT might explain the impaired ability to use fat stores and progression of the disease (42). Hence, there is a need for a more complex understanding of how leptin drives SNS activity than what we currently have. The activity of SNS has been described to be preserved/elevated in other tissues and lost specifically in the adipose tissue (both WAT and BAT). As such, leptin resistance could entail catecholamine resistance in the adipose tissue (43). Consistent with this idea, recent studies have shown that sympathetic neuro-adipose junctions drive local lipolysis in the adipose tissue (37). Local gain-of-function of sympathetic neuronal activity in the inguinal fat pad drives local lipolysis and local reduction of fat mass (37). Conversely, local loss-of-function of sympathetic neurons in the fat pad abrogates leptin’s lipolytic action in a local manner (37).

Taking into account all the aspects referred above, leptin resistance may also involve decreased sympathetic local activity, within the adipose organ. In this regard, and before the leptin era, George Bray and others proposed the “MONA LISA” hypothesis (*M*ost *O*besities k*N*own *A*re *L*ow *I*n *S*ympathetic *A*ctivity). This hypothesis was built up from studies of SNS activity and norepinephrine decay in obese patients and rats (44–46). However, under the light of the well-known obesity induced hypertension, which is associated with increased sympathetic drive to the vasculature and kidney, the MONA LISA hypothesis was later regarded as a paradoxical model (47). The conciliation of having low and high SNS activity in the same model was difficult to attain at a time when the molecular genetics of obesity was giving its first steps. The view that a multitude of cells has to respond in the same manner to the same stimulus, such as obesity, assumes that all SNS neurons are identical. This type of *tabula rasa* models precede the era of molecular genetics, which paved the way for molecular and functional diversity of seemingly alike cells. Indeed, the era of molecular genetics has enabled the mechanistic dissection of brain circuits, as well as, the immune system in spectacular ways. However, the molecular and circuit organization of the SNS, which innervates all known organs, is essentially unexplored. A molecular neuroanatomical map of the SNS may in turn revive the MONA LISA hypothesis. Once we understand the diversity of the SNS from a molecular and circuit standpoint, we then may be closer to resolving the mistery of leptin resistance.

## Author Contributions

All authors listed have made substantial, direct, and intellectual contribution to the work and approved it for publication.

## Conflict of Interest Statement

The authors declare that the research was conducted in the absence of any commercial or financial relationships that could be construed as a potential conflict of interest.
